# Supplementary material for: COZOID: contact zone identifier for visual analysis of protein-protein interactions
Source: BMC Bioinformatics. 2018 Apr 6;19:125. doi: 10.1186/s12859-018-2113-6 (PMC5889581; doi:10.1186/s12859-018-2113-6)
Supplement: Supplementary file 2 — Software build. Executable binary file of the software tool. (ZIP 118,784 kb) [file 12859_2018_2113_MOESM2_ESM.zip › cozoid/java/docs/beansbinding-1.2.1-doc.zip!/org/jdesktop/el/impl/parser/ELParserConstants.html]

ELParserConstants (Beans Binding)


---


|  |  |  |
| --- | --- | --- |
| **Overview** \| **Package** \| **Class** \| **Use** \| **Tree** \| **Deprecated** \| **Index** \| **Help** | |  |
| **PREV CLASS**   **NEXT CLASS** | **FRAMES**    **NO FRAMES**     **All Classes** |  |
| SUMMARY: NESTED \| FIELD \| CONSTR \| METHOD | DETAIL: FIELD \| CONSTR \| METHOD |  |


---


## org.jdesktop.el.impl.parser Interface ELParserConstants

**All Known Implementing Classes:**: ELParser, ELParserTokenManager

---

``` public interface ELParserConstants ```

---

| **Field Summary** | |
| --- | --- |
| `static int` | `AND0` |
| `static int` | `AND1` |
| `static int` | `BADLY_ESCAPED_STRING_LITERAL` |
| `static int` | `COLON` |
| `static int` | `COMMA` |
| `static int` | `DEFAULT` |
| `static int` | `DIGIT` |
| `static int` | `DIV0` |
| `static int` | `DIV1` |
| `static int` | `DOT` |
| `static int` | `EMPTY` |
| `static int` | `END_EXPRESSION` |
| `static int` | `EOF` |
| `static int` | `EQ0` |
| `static int` | `EQ1` |
| `static int` | `EXPONENT` |
| `static int` | `FALSE` |
| `static int` | `FLOATING_POINT_LITERAL` |
| `static int` | `FUNCTIONSUFFIX` |
| `static int` | `GE0` |
| `static int` | `GE1` |
| `static int` | `GT0` |
| `static int` | `GT1` |
| `static int` | `IDENTIFIER` |
| `static int` | `ILLEGAL_CHARACTER` |
| `static int` | `IMPL_OBJ_START` |
| `static int` | `IN_EXPRESSION` |
| `static int` | `INSTANCEOF` |
| `static int` | `INTEGER_LITERAL` |
| `static int` | `LBRACK` |
| `static int` | `LE0` |
| `static int` | `LE1` |
| `static int` | `LETTER` |
| `static int` | `LITERAL_EXPRESSION` |
| `static int` | `LPAREN` |
| `static int` | `LT0` |
| `static int` | `LT1` |
| `static int` | `MINUS` |
| `static int` | `MOD0` |
| `static int` | `MOD1` |
| `static int` | `MULT` |
| `static int` | `NE0` |
| `static int` | `NE1` |
| `static int` | `NOT0` |
| `static int` | `NOT1` |
| `static int` | `NULL` |
| `static int` | `OR0` |
| `static int` | `OR1` |
| `static int` | `PLUS` |
| `static int` | `QUESTIONMARK` |
| `static int` | `RBRACK` |
| `static int` | `RPAREN` |
| `static int` | `START_DEFERRED_EXPRESSION` |
| `static int` | `START_DYNAMIC_EXPRESSION` |
| `static int` | `STRING_LITERAL` |
| `static java.lang.String[]` | `tokenImage` |
| `static int` | `TRUE` |

**Field Detail**

### EOF

```
static final int EOF
```

**See Also:**: Constant Field Values

---


### LITERAL\_EXPRESSION

```
static final int LITERAL_EXPRESSION
```

**See Also:**: Constant Field Values

---


### START\_DYNAMIC\_EXPRESSION

```
static final int START_DYNAMIC_EXPRESSION
```

**See Also:**: Constant Field Values

---


### START\_DEFERRED\_EXPRESSION

```
static final int START_DEFERRED_EXPRESSION
```

**See Also:**: Constant Field Values

---


### INTEGER\_LITERAL

```
static final int INTEGER_LITERAL
```

**See Also:**: Constant Field Values

---


### FLOATING\_POINT\_LITERAL

```
static final int FLOATING_POINT_LITERAL
```

**See Also:**: Constant Field Values

---


### EXPONENT

```
static final int EXPONENT
```

**See Also:**: Constant Field Values

---


### STRING\_LITERAL

```
static final int STRING_LITERAL
```

**See Also:**: Constant Field Values

---


### BADLY\_ESCAPED\_STRING\_LITERAL

```
static final int BADLY_ESCAPED_STRING_LITERAL
```

**See Also:**: Constant Field Values

---


### TRUE

```
static final int TRUE
```

**See Also:**: Constant Field Values

---


### FALSE

```
static final int FALSE
```

**See Also:**: Constant Field Values

---


### NULL

```
static final int NULL
```

**See Also:**: Constant Field Values

---


### END\_EXPRESSION

```
static final int END_EXPRESSION
```

**See Also:**: Constant Field Values

---


### DOT

```
static final int DOT
```

**See Also:**: Constant Field Values

---


### LPAREN

```
static final int LPAREN
```

**See Also:**: Constant Field Values

---


### RPAREN

```
static final int RPAREN
```

**See Also:**: Constant Field Values

---


### LBRACK

```
static final int LBRACK
```

**See Also:**: Constant Field Values

---


### RBRACK

```
static final int RBRACK
```

**See Also:**: Constant Field Values

---


### COLON

```
static final int COLON
```

**See Also:**: Constant Field Values

---


### COMMA

```
static final int COMMA
```

**See Also:**: Constant Field Values

---


### GT0

```
static final int GT0
```

**See Also:**: Constant Field Values

---


### GT1

```
static final int GT1
```

**See Also:**: Constant Field Values

---


### LT0

```
static final int LT0
```

**See Also:**: Constant Field Values

---


### LT1

```
static final int LT1
```

**See Also:**: Constant Field Values

---


### GE0

```
static final int GE0
```

**See Also:**: Constant Field Values

---


### GE1

```
static final int GE1
```

**See Also:**: Constant Field Values

---


### LE0

```
static final int LE0
```

**See Also:**: Constant Field Values

---


### LE1

```
static final int LE1
```

**See Also:**: Constant Field Values

---


### EQ0

```
static final int EQ0
```

**See Also:**: Constant Field Values

---


### EQ1

```
static final int EQ1
```

**See Also:**: Constant Field Values

---


### NE0

```
static final int NE0
```

**See Also:**: Constant Field Values

---


### NE1

```
static final int NE1
```

**See Also:**: Constant Field Values

---


### NOT0

```
static final int NOT0
```

**See Also:**: Constant Field Values

---


### NOT1

```
static final int NOT1
```

**See Also:**: Constant Field Values

---


### AND0

```
static final int AND0
```

**See Also:**: Constant Field Values

---


### AND1

```
static final int AND1
```

**See Also:**: Constant Field Values

---


### OR0

```
static final int OR0
```

**See Also:**: Constant Field Values

---


### OR1

```
static final int OR1
```

**See Also:**: Constant Field Values

---


### EMPTY

```
static final int EMPTY
```

**See Also:**: Constant Field Values

---


### INSTANCEOF

```
static final int INSTANCEOF
```

**See Also:**: Constant Field Values

---


### MULT

```
static final int MULT
```

**See Also:**: Constant Field Values

---


### PLUS

```
static final int PLUS
```

**See Also:**: Constant Field Values

---


### MINUS

```
static final int MINUS
```

**See Also:**: Constant Field Values

---


### QUESTIONMARK

```
static final int QUESTIONMARK
```

**See Also:**: Constant Field Values

---


### DIV0

```
static final int DIV0
```

**See Also:**: Constant Field Values

---


### DIV1

```
static final int DIV1
```

**See Also:**: Constant Field Values

---


### MOD0

```
static final int MOD0
```

**See Also:**: Constant Field Values

---


### MOD1

```
static final int MOD1
```

**See Also:**: Constant Field Values

---


### IDENTIFIER

```
static final int IDENTIFIER
```

**See Also:**: Constant Field Values

---


### FUNCTIONSUFFIX

```
static final int FUNCTIONSUFFIX
```

**See Also:**: Constant Field Values

---


### IMPL\_OBJ\_START

```
static final int IMPL_OBJ_START
```

**See Also:**: Constant Field Values

---


### LETTER

```
static final int LETTER
```

**See Also:**: Constant Field Values

---


### DIGIT

```
static final int DIGIT
```

**See Also:**: Constant Field Values

---


### ILLEGAL\_CHARACTER

```
static final int ILLEGAL_CHARACTER
```

**See Also:**: Constant Field Values

---


### DEFAULT

```
static final int DEFAULT
```

**See Also:**: Constant Field Values

---


### IN\_EXPRESSION

```
static final int IN_EXPRESSION
```

**See Also:**: Constant Field Values

---


### tokenImage

```
static final java.lang.String[] tokenImage
```


---


|  |  |  |
| --- | --- | --- |
| **Overview** \| **Package** \| **Class** \| **Use** \| **Tree** \| **Deprecated** \| **Index** \| **Help** | |  |
| **PREV CLASS**   **NEXT CLASS** | **FRAMES**    **NO FRAMES**     **All Classes** |  |
| SUMMARY: NESTED \| FIELD \| CONSTR \| METHOD | DETAIL: FIELD \| CONSTR \| METHOD |  |


---
